# Draft Genome Sequence of Lawsonibacter asaccharolyticus JCM 32166^T^, a Butyrate-Producing Bacterium, Isolated from Human Feces

**DOI:** 10.1128/genomeA.00563-18

**Published:** 2018-06-21

**Authors:** Mitsuo Sakamoto, Nao Ikeyama, Masahiro Yuki, Moriya Ohkuma

**Affiliations:** a Microbe Division/Japan Collection of Microorganisms, RIKEN BioResource Research Center, Tsukuba, Japan; b PRIME, Japan Agency for Medical Research and Development (AMED), Tsukuba, Japan

## Abstract

Here, we report the draft genome sequence of Lawsonibacter asaccharolyticus JCM 32166^T^, a butyrate-producing bacterium, isolated from human feces. The genomic analysis reveals genes for butyrate synthesis and will facilitate the study on the role of this strain in the human gut.

## GENOME ANNOUNCEMENT

Human gut microbiome research has become increasingly important. During our attempts to recover microbes with beneficial properties, such as butyrate-producing bacteria or flavonoid-degrading bacteria, we have successfully isolated a butyrate-producing bacterium, designated strain 3BBH22^T^ (= JCM 32166^T^), from a feces sample from a healthy Japanese woman. Recently, strain 3BBH22^T^ was proposed to belong to a novel species in a novel genus of the family Ruminococcaceae, for which the name Lawsonibacter asaccharolyticus was given (1). Members of Lachnospiraceae and Ruminococcaceae have received the most attention (2) because they are very abundant in the human colon, comprising 10 to 20% of the total bacteria. There are four main pathways known for butyrate production, the acetyl-coenzyme A (acetyl-CoA), glutarate, 4-aminobutyrate, and lysine pathways (3). We analyzed the draft genome sequence of L. asaccharolyticus JCM 32166^T^ to elucidate the mechanisms of the butyrate synthesis pathway.

Chromosomal DNA was extracted from L. asaccharolyticus JCM 32166^T^ using a Genomic-tip 100/G kit (Qiagen). Labiase (5.0 mg/ml; Cosmo Bio) was used to lyse bacterial cells. The whole genome of L. asaccharolyticus JCM 32166^T^ was sequenced using the PacBio RS II sequencing system (Pacific Biosciences) by TaKaRa. The reads were assembled *de novo* using Hierarchical Genome Assembly Process version 3.0 (HGAP3.0) in SMRT Analysis version 2.3.0 (4), resulting in 7 contigs with an *N*_50_ length of 3,503,692 bp. This assembly resulted in a draft genome sequence of 4,282,156 bp, with a G+C content of 58.4%. Analysis of the genome sequences was performed using the Microbial Genome Annotation Pipeline (MiGAP; https://www.migap.org/index.php/en) (5). A total of 4,461 protein-coding sequences (CDSs), 85 tRNAs, and 6 rRNAs were detected.

As expected, the genome of L. asaccharolyticus JCM 32166^T^ contained an acetyl-CoA acetyltransferase (AtoB or Thl; EC 2.3.1.9) gene, 3-hydroxybutyryl-CoA dehydrogenase (Hbd; EC 1.1.1.157) gene, 3-hydroxybutyryl-CoA dehydratase (Crt; EC 4.2.1.55) gene, butyryl-CoA dehydrogenase (Bcd; EC 1.3.8.1, including electron transfer flavoprotein α,β-subunits; FixB and FixA) gene, phosphate butyryltransferase (Ptb; EC 2.3.1.19) gene, and butyrate kinase (Buk; EC 2.7.2.7) gene, verifying that it possesses the acetyl-CoA pathway (3). Although Intestinimonas butyriciproducens (6), which is phylogenetically related to L. asaccharolyticus JCM 32166^T^ (1), possesses the lysine pathway (7, 8), L. asaccharolyticus JCM 32166^T^ did not. It has been reported that the acetyl-CoA pathway is the most prevalent, followed by the lysine pathway (3). In this strain, butyrate is produced via Buk, leading to the formation of ATP. The genome sequence will facilitate further studies on the beneficial role of this strain in the human gut.

### Accession number(s).

The draft genome sequence of L. asaccharolyticus JCM 32166^T^ has been deposited in DDBJ/EMBL/GenBank under the accession numbers BFBT01000001 to BFBT01000007.
